# Colloid Metals in Therapeutics

**Published:** 1914-05-02

**Authors:** 


### COLLOID METALS IN THERAPEUTICS.

In another part of this issue (see p. 125) will be found an article which sounds a much-needed note of warning concerning the use and abuse of colloid metals. For details of the diseases for which these substances are being just now largely prescribed—and lustily boomed by firms interested in their sale and manufacture—the article itself may be consulted; for it represents not only the actual experience of the author, a practising clinician, but also a careful study of the literature and first-hand inquiries into the results obtained institutionally. It would appear that during the last few months the colloid metals have been elevated to the unenviable notoriety of the latest "fad" in the therapeutical world. The result is that practitioners of the pushful and not over-scrupulous type are experimenting with colloid metals as a kind of panacea for all human ills. The bulk of the medical profession, when situations of this sort arise, as they not uncommonly do, is faced with a difficult problem. If practitioners hang back, await reports from trustworthy sources, and refuse to countenance colloid therapy in any and every serious disease, they may get a reputation with the public for being "out-of-date," ultra-conservative, or simply stupidly ignorant. If, on the other hand, they adopt the much easier plan of swimming with the current, that is, of experimenting right and left with these potent and dangerous drugs as long as the craze for them lasts, they are not doing what is right for their patients, though they may gain in popularity and in pocket.

The indiscriminating employment of colloid metals for all kinds of diseases, and especially for cancer (whether operable or not) will certainly result in so many disappointments that the colloids will speedily get a bad name and disappear from the list of permissible remedies. This is all the more to be deprecated, inasmuch as it seems quite possible that a real use will be found for the colloids in certain septic cases. It were greatly to be desired that some authoritative body should entrust to a committee of capable and reputable scientific physicians and surgeons an investigation which would result in an unbiassed report setting forth the pros and cons of colloid therapy. As matters stand, only the optimistic reports get published, and they emanate chiefly from practitioners who have been equally enthusiastic in times past about some one or more of the discarded crazes that have been fashionable in recent years. There is reason to believe that a great deal of harm is being done by colloid treatment in unsuitable cases, and that actual cases of poisoning have occurred which have never been reported in the medical press.

